# Early Change as a Predictor of Treatment Outcome in Patients with a Personality Disorder

**DOI:** 10.1007/s10488-024-01401-2

**Published:** 2024-08-07

**Authors:** Pauline D. Janse, Sophie Vercauteren, Rianne Weggemans, Bea G. Tiemens

**Affiliations:** 1 Pro Persona Research, Wolfheze, The Netherlands; 2 Behavioural Science Institute, Radboud University, Nijmegen, The Netherlands

**Keywords:** Early change, Predictor, Outcome measure, Personality disorder

## Abstract

A significant proportion of patients with a personality disorder do not benefit from treatment. Monitoring treatment progress can help adjust ineffective treatments. This study examined whether early changes in symptoms and personality dysfunction during the first phase of therapy could predict treatment outcomes. Data from 841 patients who received specialized treatment for personality disorders were analyzed. The study focused on whether changes in the Outcome Questionnaire-45.2 (OQ-45.2) symptom distress scale (SD), the General Assessment of Personality Disorder (GAPD), and Severity Indices of Personality Problems (SIPP) in the early phase of therapy predicted post-treatment personality dysfunction, as measured by the SIPP and GAPD. Early changes within a specific SIPP domain were the strongest predictors of post-treatment outcomes in that same domain. Early changes in symptoms significantly predicted outcomes in Self-Control, Relational Functioning, and Identity Integration, while the GAPD predicted outcomes in Self-Control and Social Attunement on the SIPP. For the GAPD, early changes on the GAPD itself, followed by early changes on the OQ-45 SD and the SIPP domain Social Attunement, were significant predictors. Thus, when it comes to personality dysfunction, early changes in a specific domain or measure are the best predictors of outcomes in that same domain. While the OQ-45 predicted some aspects of personality dysfunction, it should not replace disorder-specific measures. Additionally, the SIPP domains and the GAPD should not be used interchangeably to predict each other. In sum, considering these factors, monitoring early change can be useful in assessing progress in the treatment of patients with personality disorders.

**Supplementary Information:**

The online version contains supplementary material available at 10.1007/s10488-024-01401-2.

## Introduction

While psychotherapy has proven effective in treating patients with a personality disorder (Cristea et al., 2017; Rameckers et al., 2021; Storebø et al., 2020), there remains a pressing need for improvement in its application. For instance, a recent meta-analysis focusing on borderline personality disorder revealed that nearly half of the patients do not respond adequately to psychotherapy (Liechsenring et al., 2024). Another challenge lies in the premature discontinuation of treatment, with 20 to 33% of patients dropping out early (Dixon & Linardon, 2020; Iliakis et al., 2021; Swift & Greenberg, 2014). Consequently, a substantial proportion of patients do not benefit from treatment.

Unfortunately, psychotherapists seem to have difficulty predicting which treatments will stagnate or worsen (Hannan et al., 2005; Hatfield et al., 2010) and overestimate their effectiveness (Walfish et al., 2012). Because of this bias, there is a risk that patients will continue to be treated in the same way for too long, even when a treatment is ineffective or symptoms worsen. However, adding progress feedback to treatments through Routine Outcome Monitoring (ROM) can help improve treatment outcomes. Progress feedback is the systematic measurement of the treatment process and progress using measurement instruments (De Jong et al., 2021). Progress feedback through ROM typically uses general assessment instruments, such as the Outcome Questionnaire-45.2 (OQ-45.2; Lambert et al., 2004), which can be used in various patient groups. Disorder-specific measurement instruments, such as a questionnaire measuring only depressive symptoms, are also used. Recent meta-analyses show that the use of ROM can have positive effects on symptom reduction, particularly with clients who fail to progress, i.e., clients who are not on track (NOT) (De Jong et al., 2021; Rognstad et al., 2023). However, limited knowledge exists regarding the use of progress feedback with patients diagnosed with a personality disorder. The findings from a study by De Jong et al. (2018) suggest that it may not be universally beneficial for all types of personality disorders, indicating a need for additional research to explore this further.

Progress feedback is used to intervene as early as possible in treatment when it is not on track. Research has indicated that initial improvement in symptoms (*early change*) predicts overall treatment outcome, especially in patients with anxiety and mood disorders (Lutz et al., 2009, 2014; Schibbe et al., 2014). Early change is seen as an indicator of success and is, therefore, meaningful in clinical practice (Schibbe et al., 2014; Tiemens et al., 2016, 2020).

To measure early change, measurement tools that are sensitive to change are required. In anxiety, mood, and eating disorders, disorder-specific questionnaires seem to be more sensitive to change and, therefore, better able to measure early change and predict treatment outcomes than general questionnaires (Dingemans & Furth, 2017; Nugter et al., 2017; Schibbe et al., 2014; Van Der Mheen et al., 2018). Because disorder-specific questionnaires measure the degree of dysfunction and not symptoms in patients with a personality disorder, it is important to determine whether the difference between general and disorder-specific instruments also occurs in the case of personality disorders.

This study aimed to determine whether early changes in symptoms as measured with a general questionnaire and personality dysfunction as measured with specific questionnaires in the treatment of patients with a personality disorder would predict personality dysfunction post-treatment.

## Method

### Design and Setting

A cohort study was conducted using data from patients with a personality disorder who attended treatment at the Pro Persona mental health institution within the Center for Psychotherapy (CfP). The primary dependent variable was the level of personality disfunction as measured by the domains on the Severity Indices of Personality Problems (SIPP); the secondary treatment outcome was the level of personality disfunction as measured by the General Assessment of Personality Disorder (GAPD). The independent variables were changes on the SIPP, GAPD and in general symptomatology as measured by the Outcome Questionnaire 45.2 symptom distress scale.

The CfP (which was closed in July 2022 due to financial factors) was a third line, in- and outpatient treatment center for patients with a personality disorder. The treatment team consisted of psychiatrists, clinical psychologists, psychotherapists, health psychologists, art therapists, psychomotor therapists, system therapists, and socio-therapists.

### Patients

Patients were adults aged 18 and older with a personality disorder as the main diagnosis. They attended 2- or 4-day group treatment between September 2017 and March 2022 for 30 to 36 weeks. Within the CfP, there were two treatment clusters with a psychodynamic or a schema-therapeutic orientation. Within the psychodynamic treatment cluster, predominantly patients with internalizing personality problems were treated. In the schema-therapeutic treatment cluster, patients with predominantly externalizing personality problems were treated. At intake, diagnoses and treatment recommendations were made based on each patient’s history, the views of the patient’s family or good friend(s), and psychological test results. The main difference between the two treatment clusters, besides the difference in orientation, was the extent to which therapeutic pressure on patients could be increased. For example, the therapeutic treatment climate within the schema-therapy cluster was more structured, with an aim of reducing pressure on patients with more externalizing personality problems.

### Measures

The Severity Indices of Personality Problems (SIPP; Verheul et al., 2008)) is a self-report personality questionnaire that focuses on personality traits present in all personality disorders. It consists of 16 facets divided into five domains: *Social Attunement, Relational Functioning, Identity Integration, Responsibility, and Self-Control*.

On the SIPP, no total score is calculated, but scores on subscales indicate the severity of personality disfunction in the six domains. High scores indicate a more adaptive and better functioning personality. Items are rated on a 4-point Likert scale (1 = completely disagree, 2 = partially disagree, 3 = partially agree, and 4 = completely agree). The SIPP has two versions, a diagnostic version (SIPP-118) and an abbreviated version consisting of 60 items (SIPP-SF). Items on the SIPP-SF were derived from the long SIPP version. At intake, the diagnostic version (SIPP-118) was administered. The abbreviated version (SIPP-SF) was administered at the interim assessment (during treatment) and at the final assessment (at the end of treatment) and was used as a treatment outcome measure. The reliability and validity of the SIPP-118 are adequate to good (Feenstra et al., 2011; Verheul et al., 2008). The internal consistency of the SIPP-SF is good with Cronbach’s alpha between .81 and .88 (Rossi et al., 2017). The SIPP-118 is sensitive to measuring changes in personality aspects during treatment (Feenstra et al., 2011).

The GAPD (Berghuis & Livesley, 2022) is a self-report questionnaire that measures core components of personality dysfunction and is based on Livesley’s (2003) Adaptive Failure model. The main scales of the GAPD are strongly related to the Self and Interpersonal Functioning elements of the alternative DSM-5 model of personality disorders (APA, 2013). The total score can be used to express overall personality dysfunction. Unlike the SIPP, high scores on the GAPD reflect greater personality dysfunction. Items are rated on a 5-point Likert scale (1 = completely disagree, 2 = disagree, 3 = neutral, 4 = agree, 5 = completely agree). As with the SIPP, at intake in this study we used the long, diagnostic version (83 items) of the GAPD. The abbreviated version, the GAPD-SF (28 items), was administered at the intermediate and post-treatment. Items on the GAPD-SF were derived from the long version of the GAPD. Research on the psychometric characteristics of the GAPD that included four Dutch and Canadian samples, showed that the GAPD has adequate internal consistency (α ranged between .78 and .98) and good test–retest reliability (*r* ranged between .89 and .96 for the main scales) and discriminated between patients with and patients without a personality disorder (Berghuis & Livesley, 2022; Berghuis et al., 2013). Research on the validity of the GAPD is ongoing. Regarding convergent validity, moderate strong correlations were found between GAPD and SIPP-118 (Berghuis & Livesley, 2022).

*General symptomatology*. The Outcome Questionnaire 45.2 (OQ-45.2) is a self-report questionnaire consisting of 45 items that ask about how the patient has felt in the past week (Lambert et al., 2004). The OQ-45.2 measures several domains across three subscales: *Symptomatic Distress* (SD), *Interpersonal Relationships* (IR), and *Social Role* (SR). A total score is calculated by summing all the items. Items are rated on a 5-point Likert scale (0 = never, 1 = sometimes, 2 = rarely, 3 = often, 4 = almost always). The reliability of the Dutch version of the questionnaire is good, its validity is adequate and there is a high sensitivity to change on all subscales (De Jong et al., 2008). In the current study only the SD-scale was used, which of the subscales has the highest internal consistency and discriminates between a normal and clinical population (Timman et al., 2017) and is the most sensitive to change (De Beurs et al., 2019).

### Procedure

As part of the treatment at CfP, patients’ progress was monitored through ROM, and the ROM trajectory was linked to scheduled evaluation times. Data consisted of the ROM scores of 841 patients from September 2017 to March 2022. The outcome of the pre-treatment, intermediate, and post-treatment scores on the SIPP-118, SIPP-SF, OQ-45.2, GAPD, and GAPD-SF were used in this study. Patients who objected to their data being used could report this to the Care Monitoring Department of Pro Persona, after which they were excluded from the study. All patients’ data were anonymized according to the k-anonymity method (Sweeney, 2002) before being made available for the study.

### Definition of Early Change

In previous studies of the predictive value of early change in the treatment of patients with an anxiety or a mood disorder (e.g., Schibbe et al., 2014), *early* was often defined as sometime during the first half of treatment, and this rule was also followed in this study. Assessment times were defined as follows:

- The initial assessment was undertaken before the intake appointment and as close as possible to the start of treatment.
- The early change was measured at two time points: the first measurement fell between 2 and 8 weeks, and the second between 8 and 15 weeks.
- The final assessment was taken between the 4 weeks before and the 4 weeks after treatment ended.

### Statistical analyses

G*Power 3.1 (Faul et al., 2009) was used to calculate the number of participants needed. With a medium effect size of 0.10 (for multiple regression), an α of 0.05, and a power of 0.80, the required sample size was 151. A medium effect size was estimated based on previous studies (Schibbe et al., 2014).

Before running the main analyses patterns of missing data were analyzed (using the VIM package for R) and missing data was imputed using a machine learning-based data imputation algorithm that operates on the Random Forest algorithm. The advantage of this method is that it can handle non-linear and interaction effects. After imputation scores on the measures were converted to z-scores and assumptions for regression were checked, including investigating residual plots and a curve estimation. Furthermore, distributions of the diagnoses over treatment types and frequency were tested using a chi-square test, correlations between measures were explored and pre-post cohen’s *d* effect sizes for on all the measures were calculated.

Early change, the change that occurred in the first half of treatment, was measured in three ways: Early change in symptoms measured by participants’ scores on the SD subscale of the OQ-45.2; early change in personality dysfunction measured on the SIPP-SF domains and scores on the GAPD. As described above, early change was measured at two time points. The outcome measures, or dependent variables, were residualized post-treatment scores on the SIPP and the GAPD, case-mix corrected for the following covariates: age, gender, pre-treatment scores on all the measures, treatment frequency (2- or 4-day treatment), and treatment type (a psychodynamic or a schema-therapeutic orientation).

Multiple regression analyses were performed to compare the predictive value of early change in general and early change in symptoms and personality functioning. Because SIPP as an outcome measure could not be calculated as a mean total score, analyses were conducted separately for each domain. In each regression analysis, in the first model, the early change on the same domain/ measure as the dependent variable was entered; in the second model the other SIPP domains, GAPD and OQ-45 SD were added. Separate analyses were performed for the two intermediate time points. The standardized beta (β) was used to compare the variables in the second models to see which had the strongest relationship with the dependent variable. Due to the multiple comparisons a Bonferroni correction of the significance level was used to protect against a Type I error. The *p* value was set at .005.

Analyses were performed in R and SPSS 29. The R package VIM was used for the analysis of missing data and missForest for imputation of missing data.

### Missing Data

There was no missing data on the covariates age, gender, treatment frequency, and treatment type. However, there was missing data on the OQ-45, GAPD and SIPP. The percentage of missing data varied between 23.1 and 68.7%, depending on the measurement instrument used (see Table 1 of the Supplementary materials for the percentages for each measure and time point). Analysis of the missing data indicated that the data were Missing at Random (MAR; see the Supplementary material for further information). A random forest algorithm (missForest) was used to impute the missing data using all the variables: the patient’s age, gender, diagnosis, treatment frequency and type and scores on the all the measures. The missForest package has shown to perform well for a large percentage of missing values (Gómez-Méndez & Joly, 2023). The normalized root mean squared error (NRMSE) gives an indication of the model’s performance, with values close to 0 indicating good performance and values around 1 indicating bad performance (Stekhoven & Bühlmann, 2012; Stekhoven, 2022). The NRMSE values for this imputation ranged between .02 and .12. Table 2 of the Supplementary materials report the means and standard deviations of the imputed versus the original data.

## Results

### Descriptive Statistics

Table 1 shows the demographic and clinical characteristics of the 841 patients. The difference in the distributions of the diagnoses over treatment types (psychodynamic and schema therapy) were significant (*χ*^2^ (4, *N* = 764) = 162.45, *p* < .001, with almost all (94%) of the patients with borderline personality disorder treated in the schema therapy cluster. The difference in the distributions of the diagnoses over treatment frequency was not significant (*χ*^2^ (4, *N* = 764) = 8.51, *p* = .074).

**Table 1:** Demographic and clinical characteristics

Variables	Category	*N* (%)
Age category	18–25	279 (33.2%)
	26–35	296 (35.2%)
	36–45	154 (18.3%)
	46–55	90 (10.7%)
	56+	22 (2.6%)
Gender	Male	239 (27.3%)
	Female	611 (72.7%)
Diagnosis	Borderline personality disorder	135 (16.1%)
	Obsessive compulsive personality disorder	28 (3.3%)
	Avoidant personality disorder	236 (28.1%)
	Dependent personality disorder	6 (.7%)
	Other specified personality disorder	359 (42.7%)
	Missing	77 (9.2%)
Treatment frequency	2-day	309 (36.7%)
	4-day	532 (63.3%)
Treatment type	Psychodynamic	424 (50.4%)
	Schema therapy	417 (49.6%)

Overall, treatments were effective, with pre-post Cohen’s *d* effect sizes ranging between *d* = .39 and *d* = . 1.07 on the SIPP domains, *d* = .88 on the OQ-45 SD scale and *d* = .54 on the GAPD.

### Early Change as Predictive of Personality Dysfunction

**Fig. 1:**
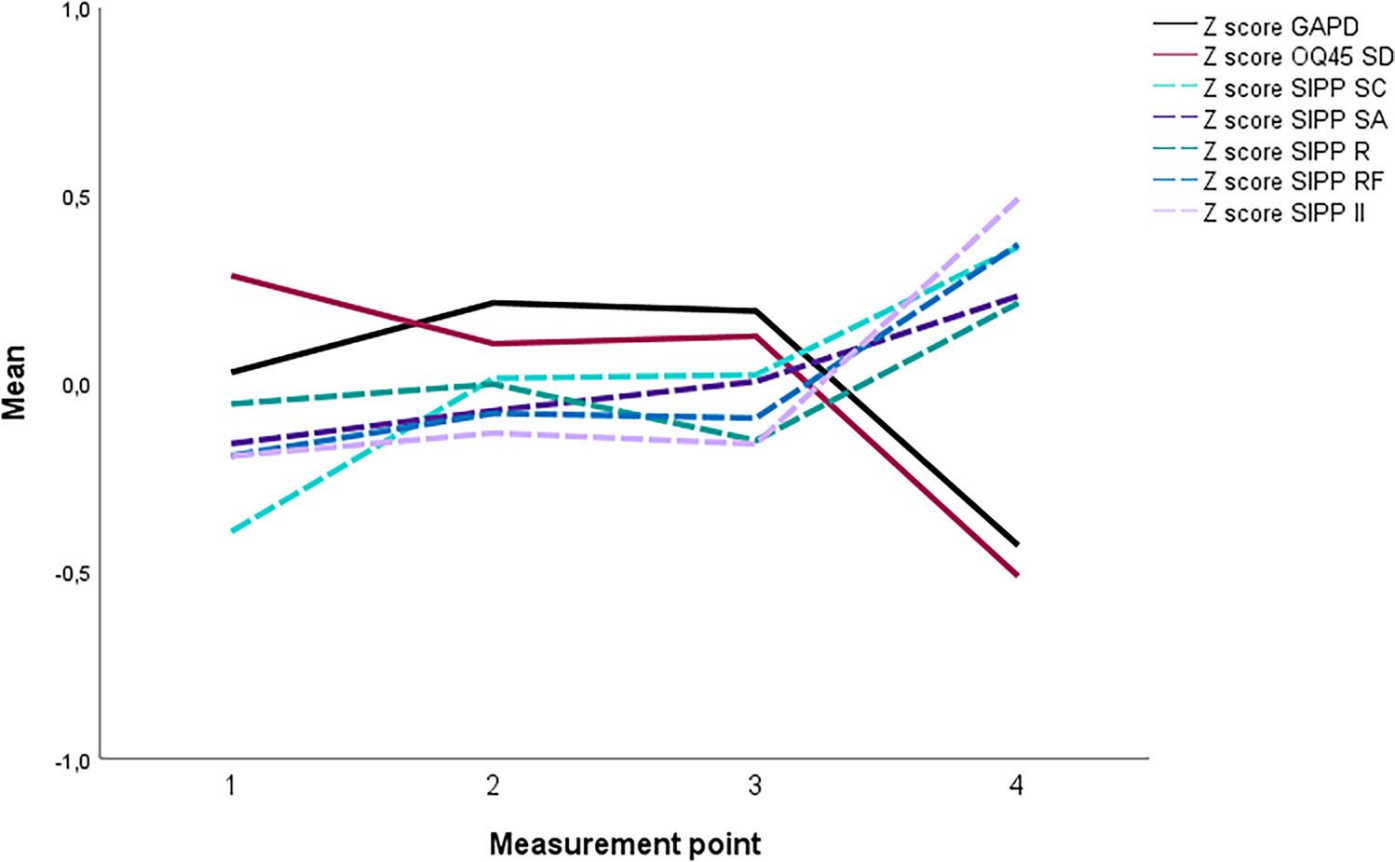
Z scores on the GADP, OQ-45 SD and SIPP domains at four measurement points. *GAPD* General Assessment of Personality Disorder, *OQ-45.2, SD* Outcome Questionniare-45.2, Symptomatic Distress scale, *SIPP* Severity Indices of Personality Problems, *SC* self-control, *SA* social attunement, *R* responsibility, *RF* relational functioning, *II* identity integration

First the data was explored by viewing the z-scores on the measures (see Fig. 1) and the correlations between the measures (see Table 3 of the Supplementary materials). We ran a curve estimation to check for possible curvilinear relationships, which showed that although the relationships between independent and dependent variables in our models were linear, there was one exception, namely early change on Social attunement at measurement time 2, which showed a curvilinear relationship. Thus, a quadratic term was added to this model.

Tables 2 and 3 show the results of the multiple regression analyses with the early change at the two intermediate time points on the separate SIPP domains, GAPD, and OQ-45 SD scales as predictors of the residualized post-treatment scores on the SIPP domains and GAPD scores. No indications of multicollinearity were found in any of the regression analyses, with the tolerance ranging between .61 and 1 and the VIF between 1.21 and 1.63.

**Table 2:** Results of Regression analyses of the effect of early change on SIPP post-treatment scores

Domain: Self-control (SC), early change measurement 1								
		B	*SE*	*β*	*t*	*p*	95.0%	CI
1	(Constant)	0	.18		0	1.000	− .36	.36
	SIPP SC early change	2.63	.18	.44	14.39	< .001	2.27	2.99
2	(Constant)	0	.18		0	1.000	− .34	.34
	SIPP SC early change	1.96	.21	.33	9.54	< .001	1.55	2.36
	SIPP SA early change	.45	.20	.08	2.27	.023	.06	.85
	SIPP R early change	.47	.19	.08	2.46	.014	.10	.85
	SIPP RF early change	− .49	.20	− .08	− 2.43	.016	− .89	− .09
	SIPP II early change	− .27	.22	− .04	− 1.20	.232	− .70	.17
	GAPD early change	.87	.21	.15	4.13	< .001	.45	1.28
	OQSD early change	1.03	.21	.17	4.91	< .001	.62	1.44
Model 1 R^2^ change = .198*; Model 2 R^2^ change = .067*. Total R^2^ = .265 (Adjusted R^2^ = .259); *p ≤ .001								

Domain: Self-control (SC), early change measurement 2								
		B	*SE*	*β*	*t*	*p*	95.0%	CI
1	(Constant)	0	.18		0	1.000	− .36	.36
	SIPP SC early change	2.66	.18	.45	14.59	< .001	2.30	3.02
2	(Constant)	0	.18		0	1.000	− .35	.35
	SIPP SC early change	2.04	.21	.35	9.89	< .001	1.64	2.44
	SIPP SA early change	.46	.20	.08	2.24	.025	.06	.86
	SIPP R early change	.50	.20	.08	2.52	.012	.11	.88
	SIPP RF early change	− .43	.21	− .07	− 2.07	.039	− .84	− .02
	SIPP II early change	− .14	.23	− .02	− .63	.529	− .59	.30
	GAPD early change	.67	.21	.11	3.13	.002	.25	1.09
	OQSD early change	.80	.21	.13	3.73	< .001	.38	1.21
Model 1 R^2^ change = .202*; Model 2 R^2^ change = .049*. Total R^2^ = .251 (Adjusted R^2^ = .245); * *p* = < .001								

Domain: Social attunement (SA), early change measurement 1								
		B	*SE*	*β*	*t*	*p*	95.0%	CI
1	(Constant)	0	.16		0	1.000	− .32	.32
	SIPP SA early change	3.13	.16	.55	19.29	< .001	2.81	3.45
2	(Constant)	0	.16		0	1.000	− .31	.31
	SIPP SA early change	2.80	.18	.50	15.50	< .001	2.45	3.15
	SIPP SC early change	.16	.18	.03	.86	.388	− .20	.52
	SIPP R early change	.38	.17	.07	2.17	.030	.04	.72
	SIPP RF early change	− .39	.18	− .07	− 2.11	.036	− .75	− .03
	SIPP II early change	− .03	.20	.00	− .14	.890	− .42	.37
	GAPD early change	.77	.19	.14	4.08	< .001	.40	1.14
	OQSD early change	.45	.19	.08	2.38	.018	.08	.82
Model 1 R^2^ change = .307*, Model 2 R^2^ change = .038*. Total R^2^ = .345 (Adjusted R^2^ = .339); *p = < .001								

Domain: Social attunement (SA), early change measurement 2								
		B	*SE*	*β*	*t*	*p*	95.0%	CI
1	(Constant)	.20	.19		1.09	.274	− .16	.57
	SIPP SA early change	2.95	.17	.52	17.77	< .001	2.62	3.27
	SIPP SA early change sq	− .20	.09	− .07	− 2.33	.020	− .38	− .03
2	(Constant)	.20	.18		1.07	.285	− .16	.55
	SIPP SA early change	2.63	.19	.47	14.07	< .001	2.26	2.99
	SIPP SA early change sq	− .20	.09	− .07	− 2.26	.024	− .37	− .03
	SIPP SC early change	.25	.19	.04	1.34	.180	− .12	.62
	SIPP R early change	.43	.18	.08	2.38	.018	.07	.78
	SIPP RF early change	− .51	.19	− .09	− 2.69	.007	− .89	− .14
	SIPP II early change	.08	.21	.01	.39	.698	− .32	.48
	GAPD early change	.75	.20	.13	3.81	< .001	.36	1.13
	OQSD early change	.27	.19	.05	1.40	.162	− .109	.65
Model 1 R^2^ change = .286*, Model 2 R^2^ change = .037*. Total R^2^ = .323 (Adjusted R^2^ = .316); *p = < .001								

Domain: Responsibility (R), early change measurement 2								
		B	*SE*	*β*	*t*	*p*	95.0%	CI
1	(Constant)	0	.19		0	1.000	− .37	.37
	SIPP R early change	3.92	.19	.58	20.51	< .001	3.54	4.29
2	(Constant)	0	.19		0	1.000	− .37	.37
	SIPP R early change	3.55	.21	.52	17.13	< .001	3.14	3.95
	SIPP SC early change	.18	.22	.03	.82	.411	− .25	.61
	SIPP SA early change	.12	.21	.02	.54	.590	− .31	.54
	SIPP RF early change	.14	.22	.02	.65	.515	− .29	.57
	SIPP II early change	− .30	.24	− .04	− 1.25	.212	− .76	.17
	GAPD early change	.78	.22	.11	3.47	< .001	.34	1.22
	OQSD early change	.52	.22	.08	2.34	.019	.09	.96
Model 1 R^2^ change = .334*, Model 2 R^2^ change = .025*. Total R^2^ = .359 (Adjusted R^2^ = .354); *p = < .001								

Domain: Responsibility (R), early change measurement 2								
		B	*SE*	*β*	*t*	*p*	95.0%	CI
1	(Constant)	0	.19		0	1.000	− .38	.38
	SIPP R early change	3.92	.19	.58	20.48	< .001	3.54	4.29
2	(Constant)	0	.19		0	1.000	− .37	.37
	SIPP R early change	3.58	.21	.53	17.09	< .001	3.17	3.99
	SIPP SC early change	.25	.22	.04	1.14	.253	− .18	.68
	SIPP SA early change	.05	.22	.01	.23	.818	− .38	.48
	SIPP RF early change	.13	.22	.02	.56	.574	− .31	.56
	SIPP II early change	− .40	.24	− .06	− 1.66	.096	− .87	.07
	GAPD early change	.78	.23	.12	3.42	< .001	.33	1.23
	OQSD early change	.47	.23	.07	2.09	.037	.03	.92
Model 1 R^2^ change = .333*, Model 2 R^2^ change = .023*. Total R^2^ = .356 (Adjusted R^2^ = .351); * *p* = < .001								

Domain: Relational functioning (RF), early change measurement 1								
		B	*SE*	*β*	*t*	*p*	95.0%	CI
1	(Constant)	0	.20		0	1.000	− .39	.39
	SIPP RF early change	2.88	.20	.45	14.48	< .001	2.49	3.27
2	(Constant)	0	.19		0	1.000	− .38	.38
	SIPP RF early change	2.16	.22	.34	9.67	< .001	1.72	2.60
	SIPP SC early change	.28	.22	.04	1.26	.209	− .16	.72
	SIPP SA early change	.08	.22	.01	.35	.728	− .35	.51
	SIPP R early change	.53	.21	.08	2.49	.013	.11	.94
	SIPP II early change	− .14	.24	− .02	− .58	.565	− .62	.34
	GAPD early change	.55	.23	.08	2.38	.018	.10	1.00
	OQSD early change	1.08	.23	.17	4.72	< .001	.63	1.53
Model 1 R^2^ change = .200*, Model 2 R^2^ change = .057*. Total R^2^ = .256 (Adjusted R^2^ = .250); *p = < .001								

Domain: Relational functioning (RF), early change measurement 2								
		B	*SE*	*β*	*t*	*p*	95.0%	CI
1	(Constant)	0	.20		0	1.000	− .40	.40
	SIPP RF early change	2.73	.20	.42	13.53	< .001	2.33	3.12
2	(Constant)	0	.20		0	1.000	− .39	.39
	SIPP RF early change	2.02	.23	.31	8.70	< .001	1.56	2.47
	SIPP SC early change	.40	.23	.06	1.73	.083	− .05	.85
	SIPP SA early change	− .06	.23	− .01	− .27	.784	− .51	.38
	SIPP R early change	.48	.22	.07	2.20	.028	.05	.91
	SIPP II early change	.04	.25	.01	.17	.863	− .45	.54
	GAPD early change	.50	.24	.08	2.11	.035	.04	.97
	OQSD early change	.83	.24	.13	3.51	< .001	.37	1.29
Model 1 R^2^ change = .179*, Model 2 R^2^ change = .047*. Total R^2^ = .226 (Adjusted R^2^ = .219); *p = < .001								

Domain: Identity integration (II), early change measurement 1								
		B	*SE*	*β*	*t*	*p*	95.0%	CI
1	(Constant)	0	.26		0	1.000	− .51	.51
	SIPP II early change	2.62	.26	.33	10.11	< .001	2.12	3.13
2	(Constant)	0	.25		0	1.000	− .48	.48
	SIPP II early change	.98	.31	.12	3.14	.002	.37	1.59
	SIPP SC early change	.40	.29	.05	1.37	.171	− .17	.96
	SIPP SA early change	.02	.28	.00	.07	.941	− .53	.57
	SIPP R early change	.47	.27	.06	1.71	.087	− .07	1.00
	SIPP RF early change	.03	.29	.00	.12	.907	− .53	.60
	GAPD early change	.60	.30	.08	2.05	.041	.02	1.18
	OQSD early change	2.27	.29	.28	7.72	< .001	1.69	2.85
Model 1 R^2^ change = .109*, Model 2 R^2^ change = .090*. Total R^2^ = .198 (Adjusted R^2^ = .192); *p = < .001								

Domain: Identity integration (II), early change measurement 2								
		B	*SE*	*β*	*t*	*p*	95.0%	CI
1	(Constant)	0	.26		0	1.000	− .51	.51
	SIPP II early change	2.66	.26	.33	10.25	< .001	2.15	3.17
2	(Constant)	0	.25		0	1.000	− .49	.49
	SIPP II early change	1.30	.32	.16	4.07	< .001	.67	1.93
	SIPP SC early change	.54	.29	.07	1.85	.064	− .03	1.11
	SIPP SA early change	− .04	.29	− .01	− .14	.887	− .61	.53
	SIPP R early change	.43	.28	.05	1.55	.123	− .12	.98
	SIPP RF early change	− .02	.30	.00	− .06	.955	− .60	.56
	GAPD early change	.42	.30	.05	1.38	.167	− .18	1.02
	OQSD early change	1.81	.30	.23	5.99	< .001	1.22	2.40
Model 1 R^2^ change = .111*, Model 2 R^2^ change = .062*. Total R^2^ = .173 (Adjusted R^2^ = .166); *p = < .001								

*SC* self-control, *SA* social attunement, *R* responsibility, *RF* relational functioning, *II* identity integration

**Table 3:** Results of Regression analyses of the effect of early change on GAPD post-treatment

GAPD post-treatment, early change measurement 1								
		B	*SE*	*β*	*t*	*p*	95.0%	CI
1	(Constant)	0	.01		0	1.000	− .03	.03
	GAPD early change	− .16	.01	− .39	− 12.34	< .001	− .18	− .13
2	(Constant)	0	.01		0	1.000	− .02	.02
	GAPD early change	− .10	.01	− .25	− 6.74	< .001	− .13	− .07
	SIPP RF early change	− .01	.01	− .03	− .83	.408	− .04	.02
	SIPP SC early change	− .03	.01	− .07	− 1.89	.059	− .05	.00
	SIPP SA early change	− .05	.01	− .13	− 3.74	< .001	− .08	− .02
	SIPP R early change	− .01	.01	− .02	− .63	.527	− .04	.02
	SIPP II early change	.01	.02	.02	.47	.641	− .02	.04
	OQSD early change	− .09	.01	− .21	− 5.90	< .001	− .12	− .06
Model 1 R^2^ change = .154*. Model 2 R^2^ change = .073*. Total R^2^ = .226 (Adjusted R^2^ = .220); *p = < .001								

GAPD post-treatment, early change measurement 2								
		B	*SE*	*β*	*t*	*p*	95.0%	CI
1	(Constant)	0	.01		0	1.000	− .03	.03
	GAPD early change	− .16	.01	− .38	− 11.94	< .001	− .18	− .13
2	(Constant)	0	.01		0	1.000	− .02	.02
	GAPD early change	− .10	.02	− .24	− 6.32	< .001	− .13	− .07
	SIPP RF early change	− .02	.01	− .05	− 1.26	.208	− .05	.01
	SIPP SC early change	− .03	.01	− .06	− 1.81	.071	− .05	.00
	SIPP SA early change	− .05	.01	− .13	− 3.73	< .001	− .08	− .02
	SIPP R early change	.00	.01	− .01	− .18	.854	− .03	.03
	SIPP II early change	.00	.02	− .01	− .29	.768	− .04	.03
	OQSD early change	− .06	.02	− .15	− 4.16	< .001	− .09	− .03
Model 1 R^2^ change = .145*. Model 2 R^2^ change = .058*. Total R^2^ = .203 (Adjusted R^2^ = .197); *p ≤ .001								

*Note.* SC = self-control, SA = social attunement, R = responsibility, RF= relational functioning, II = identity integration

The results showed that early changes in scores on a specific SIPP domain were the strongest predictors of the residual scores on that same SIPP domain, but other SIPP domains were not (when the Bonferroni corrected *p* value of .005 is applied). The second most significant predictor for the SIPP domains of Self-Control, Relational Functioning, and Identity Integration was the early change on the OQ-45 SD. For the Self-Control domain, the third most significant predictor was the early change in the GAPD. The second strongest predictor of the SIPP domains Social attunement and Responsibility was the early change on the GAPD; on these domains the early change on the OQ-45 SD was not a significant predictor.

When the residual score on the GAPD was the outcome, the early change on the GAPD at both intermediate measurement points was the strongest predictor, followed by early changes on the OQ-45 SD scale and the SIPP domain Social Attunement, while the other SIPP domains did not predict the GAPD.

## Discussion

The primary objective of this study was to determine whether change shown early in treatment by patients with a personality disorder could predict their ultimate treatment outcome, as measured by two outcome measures, the SIPP-SF and the GAPD. A secondary aim was to determine whether the predictive value of early change depends on whether it is assessed with a general or a disorder-specific measurement instrument.

The results showed that early changes on a specific domain of the SIPP were the strongest predictors of case-mix corrected final scores on that same domain, and the proportion of explained variance ranged from 10.9 to 33.4%. This indicates that improvements or declines in specific personality domains early in treatment are highly indicative of the final outcomes in those same domains. Changes in other SIPP domains were not significant predictors of case-mix corrected final scores on a specific domain. This suggests that cross-domain predictions within SIPP are weak or non-existent. The second most significant predictor for the domains of Self-Control, Relational Functioning, and Identity Integration was the early change on the OQ-45 SD scale. This indicates that early improvements in overall symptom distress are relevant for predicting outcomes in these three personality domains. The second most significant predictor for the domains of Social Attunement and Responsibility and the third most significant predictor of the domain of Self-Control was early change in the GAPD. Thus, early changes in the general severity of personality disorder symptoms also play a role in predicting outcomes in these domains.

For the GAPD as an outcome measure, early changes in the GAPD itself at both intermediate measurement points were the strongest predictors of case-mix corrected GAPD post-treatment scores. Early changes on the OQ-45 SD scale and the SIPP domain Social Attunement were also significant predictors, indicating that improvements in overall symptom distress and Social Attunement might be relevant for predicting outcomes on the GAPD. Interestingly, in the case of the GAPD as an outcome measure, the predictive value of early change on the OQ-SD scale was stronger than that of all domains of the SIPP. In fact, early changes in the SIPP domains of Relational functioning, Identity integration, Responsibility, and Self-control were not predictive at all. The takeaway is that even though they both measure personality functioning, the SIPP domains and the GAPD cannot be used interchangeably to predict each other.

Another relevant finding was that no difference was found between the two early change moments. This means that patients with a personality disorder can also be assessed early in treatment (the first 8 weeks) whether they respond well to treatment.

As to why early changes on the OQ-45 SD predict certain SIPP domains and the GAPD but not others, this might have to do with the relationships between general symptom distress and specific personality traits. Symptom distress may have a more immediate and direct impact on domains such as Self-Control, Relational Functioning, and Identity Integration because these areas are closely connected to an individual’s emotional and psychological state. For example, in the case of the SIPP domain identity integration, the items of that domain seem to overlap with those on the OQ-45 SD scale. For instance, the items “I often see no reason to continue living” or “I often feel that I am not as worthy as other people” are similar to OQ-45 SD items “I have thoughts of ending my life” and “I feel worthless.” The highest correlation found between the OQ-45 SD and SIPP domains in the present study was with the identity integration domain (see Table 3 in the Supplementary materials). Additionally, in a study with adolescents, the correlation between the general severity index of the Symptom Checklist (SCL-90) and the identity integration domain of the SIPP was *r* = − 0.80, which is very high (Feenstra et al., 2011). The reason why early changes on the OQ-45 SD did not predict outcomes on the Social Attunement and Responsibility domain might be because these domains may rely on more stable traits or skills that are less directly impacted by general symptom distress, requiring different therapeutic approaches for improvement.

Arguably, the added explained variance of early change on other measures than the measure or domain that was the outcome variable was limited (changes in explained variance ranged from 2.3 to 9.0%), suggesting, for instance, in the case of the OQ-45 SD it cannot be relied upon to be the sole or best predictor of outcome and disorder-specific measures should always be used alongside the OQ-45 SD when monitoring treatment response with patients with personality disorders.

Furthermore, another question for future research relevant to clinical practice is how much early change is indicative of good treatment outcomes. The reliable change indices (Jacobson & Truax, 1991) for the SIPP and the GAPD may be helpful in establishing clinical guidelines for this. This will help with the interpretation of the scores. Furthermore, future research should focus more on the overlap and differences between the SIPP and GAPD, as there might measure different aspects of personality functioning or differ in their sensitivity to change, also considering that for patients, it might be burdensome to use both the SIPP and the GAPD.

There are several limitations of this study that need to be considered. The major limitation was the large percentage of missing data at various time points. We addressed this issue by using a Random Forest algorithm to impute the missing data. Imputing data is the preferred alternative to excluding participants with missing data because doing so can lead to biased results and conclusions (Madley-Dowd et al., 2019). Some of the missing data might be explained by the lack of checks to ensure the questionnaires were completed during treatment and having even less control over questionnaire completion after patients had been discharged from treatment. Additionally, the motivation of patients to complete the questionnaires might have been diminished after the termination of their treatment. Moreover, some patients transitioned to another treatment program within the same mental health organization after having received treatment in the current program (CfP), and personality questionnaires are not routinely included in the ROM in other treatment programs.

Another limitation of this study was the relatively small percentage of patients with a borderline personality disorder. This might be due to the fact that at the same mental health organization (but at other treatment locations than the CfP), Dialectical Behavior Therapy (DBT) was available as another intensive treatment option for patients with externalizing personality and severe emotion regulation problems. Therefore, it is likely that some patients with a borderline personality disorder were referred to the DBT program.

Despite these limitations, the current study is, to the best of our knowledge, the first study to examine early change in a population of patients with a personality disorder. This study showed that concerning personality dysfunction, early changes on a specific domain or measure predicted outcomes in that same domain or measure. Furthermore, early changes in symptom distress (OQ-45 SD) were significant predictors for several personality traits, especially Self-Control, Relational Functioning, Identity Integration, and the GAPD, although it had a modest effect and should not replace disorder-specific measures. In sum, in the context of personality disorder treatments, early assessments during the initial 8 weeks of inpatient care can reveal valuable insights into treatment responsiveness.

## Supplementary Information

Below is the link to the electronic supplementary material.

Supplementary file1 (DOCX 34 KB)

## References

[CR1] American Psychiatric Association. (2013). *Alternative DSM–5 model for personality disorders (DSM–5, Section III)*. American Psychiatric Publishing.

[CR3] Berghuis, H., Kamphuis, J. H., Verheul, R., Larstone, R., & Livesley, J. (2013). The General Assessment of Personality Disorder (GAPD) as an instrument for assessing the core features of personality disorders. *Clinical Psychology & Psychotherapy,**20*(6), 544–557. 10.1002/cpp.1811 22915478 10.1002/cpp.1811

[CR4] Berghuis, H., & Livesley, W. J. (2022). General Assessment of personality disorder. Vragenlijst GAPD. Kernaspecten persoonlijkheidsstoornissen. Handleiding versie mei 2022. [General Assessment of personality disorder. GAPD questionnaire. Core aspects of personality disorders. Manual version May 2022]. https://hanberghuis.nl/gapd

[CR6] Budge, S. L., Moore, J. T., Del Re, A. C., Wampold, B. E., Baardseth, T. P., & Nienhuis, J. B. (2013). The effectiveness of evidence-based treatments for personality disorders when comparing treatment-as-usual and bona fide treatments. *Clinical Psychology Review,**33*(8), 1057–1066. 10.1016/j.cpr.2013.08.003 24060812 10.1016/j.cpr.2013.08.003

[CR8] Cristea, I. A., Gentili, C., Cotet, C. D., Palomba, D., Barbui, C., & Cuijpers, P. (2017). Efficacy of psychotherapies for borderline personality disorder: A systematic review and meta-analysis. *JAMA Psychiatry,**74*(4), 319–328. 10.1001/jamapsychiatry.2016.4287 28249086 10.1001/jamapsychiatry.2016.4287

[CR9] de Beurs, E., Vissers, E., Schoevers, R., Carlier, I. V. E., van Hemert, A. M., & Meesters, Y. (2019). Comparative responsiveness of generic versus disorder-specific instruments for depression: An assessment in three longitudinal datasets. *Depression and Anxiety,**36*(1), 93–102. 10.1002/da.22809 30188602 10.1002/da.22809 PMC6586043

[CR11] de Jong, K., Conijn, J. M., Gallagher, R., Reshetnikova, A. S., Heij, M., & Lutz, M. C. (2021). Using progress feedback to improve outcomes and reduce drop-out, treatment duration, and deterioration: A multilevel meta-analysis. *Clinical Psychology Review,**85*, 102002. 10.1016/j.cpr.2021.102002 33721605 10.1016/j.cpr.2021.102002

[CR12] de Jong, K., Nugter, A., Polak, M., Wagenborg, H., Spinhoven, P., & Heiser, W. (2008). De Nederlandse versie van de Outcome Questionnaire (OQ-45): Een crossculturele validatie [The Dutch version of the Outcome Questionnaire (OQ-45): A cross-cultural validation]. *Psychologie & Gezondheid,**36*(1), 35–45. 10.1007/BF03077465 10.1007/BF03077465

[CR13] de Jong, K., Segaar, J., Ingenhoven, T., van Busschbach, J., & Timman, R. (2018). Adverse effects of outcome monitoring feedback in patients with personality disorders: A randomized controlled trial in day treatment and inpatient settings. *Journal of Personality Disorders,**32*(3), 393–413. 10.1521/pedi_2017_31_297 28594629 10.1521/pedi_2017_31_297

[CR14] Dingemans, A. E., & Van Furth, E. F. (2017). Het meten van verandering tijdens behandeling voor eetstoornissen: een vergelijking van algemene en specifieke vragenlijst. [Measuring change during treatment for eating disorders: a comparison of general and specific questionnaire]. *Tijdschrift voor psychiatrie*, *59*(5), 278–285. 28593621

[CR16] Dixon, L. J., & Linardon, J. (2020). A systematic review and meta-analysis of dropout rates from dialectical behaviour therapy in randomized controlled trials. *Cognitive Behaviour Therapy,**49*(3), 181–196. 31204902 10.1080/16506073.2019.1620324

[CR18] Faul, F., Erdfelder, E., Buchner, A., & Lang, A. G. (2009). Statistical power analyses using G* Power 3.1: Tests for correlation and regression analyses. *Behavior Research Methods,**41*(4), 1149–1160. 10.3758/BRM.41.4.1149 19897823 10.3758/BRM.41.4.1149

[CR19] Feenstra, D. J., Hutsebaut, J., Verheul, R., & Busschbach, J. J. (2011). Severity Indices of Personality Problems (SIPP–118) in adolescents: Reliability and validity. *Psychological Assessment,**23*(3), 646. 10.1037/a0022995 21500921 10.1037/a0022995

[CR20] Gómez-Méndez, I., & Joly, E. (2023). Regression with missing data, a comparison study of techniques based on random forests. *Journal of Statistical Computation and Simulation*. 10.1080/00949655.2022.2163646 10.1080/00949655.2022.2163646

[CR22] Hannan, C., Lambert, M. J., Harmon, C., Nielsen, S. L., Smart, D. W., Shimokawa, K., & Sutton, S. W. (2005). A lab test and algorithms for identifying clients at risk for treatment failure. *Journal of Clinical Psychology,**61*(2), 155–163. 10.1002/jclp.20108 15609357 10.1002/jclp.20108

[CR23] Hatfield, D., McCullough, L., Frantz, S. H., & Krieger, K. (2010). Do we know when our clients get worse? an investigation of therapists’ ability to detect negative client change. *Clinical Psychology & Psychotherapy,**17*(1), 25–32. 10.1002/cpp.656 19916162 10.1002/cpp.656

[CR24] Iliakis, E. A., Ilagan, G. S., & Choi-Kain, L. W. (2021). Dropout rates from psychotherapy trials for borderline personality disorder: A meta-analysis. *Personality Disorders: Theory, Research, and Treatment,**12*(3), 193. 10.1037/per0000453 33591777

[CR26] Jacobson, N. S., & Truax, P. (1991). Clinical significance: A statistical approach to defining meaningful change in psychotherapy research. *JCCP,**59*(1), 12–19. 10.1037//0022-006x.59.1.12 2002127

[CR28] Kowarik, A., & Templ, M. (2016). Imputation with the R Package VIM. *Journal of Statistical Software,**74*, 1–16. 10.18637/jss.v074.i07 10.18637/jss.v074.i07

[CR29] Lambert, M. J., Morton, J. J., Hatfield, D. R., Harmon, C., Hamilton, S., & Shimokawa, K. (2004). *Administration and scoring manual for the OQ-45.2 (Outcome Questionnaire)* (3rd ed.). American Professional Credentialing Services LLC.

[CR31] Leichsenring, F., Fonagy, P., Heim, N., Kernberg, O. F., Leweke, F., Luyten, P., Salzer, S., Spitzer, C., & Steinert, C. (2024). Borderline personality disorder: A comprehensive review of diagnosis and clinical presentation, etiology, treatment, and current controversies. *World Psychiatry : Official Journal of the World Psychiatric Association (WPA),**23*(1), 4–25. 10.1002/wps.21156 38214629 10.1002/wps.21156 PMC10786009

[CR32] Livesley, W. J. (2003). *Practical management of personality disorders*. Guilford Press.

[CR33] Lutz, W., Hofmann, S. G., Rubel, J., Boswell, J. F., Shear, M. K., Gorman, J. M., Woods, S. W., & Barlow, D. H. (2014). Patterns of early change and their relationship to outcome and early treatment termination in patients with panic disorder. *Journal of Consulting and Clinical Psychology,**82*(2), 287–297. 10.1037/a0035535 24447004 10.1037/a0035535 PMC3966935

[CR34] Lutz, W., Stulz, N., & Köck, K. (2009). Patterns of early change and their relationship to outcome and follow-up among patients with major depressive disorders. *Journal of Affective Disorders,**118*(1–3), 60–68. 10.1016/j.jad.2009.01.019 19217669 10.1016/j.jad.2009.01.019

[CR52] Mack, C., Su, Z., & Westreich, D. (2018). *Managing Missing Data in Patient Registries: Addendum to Registries for Evaluating Patient Outcomes: A User’s Guide* 3rd (ed.). Agency for Healthcare Research and Quality (US). 29671990

[CR35] Madley-Dowd, P., Hughes, R., Tilling, K., & Heron, J. (2019). The proportion of missing data should not be used to guide decisions on multiple imputation. *Journal of Clinical Epidemiology,**110*, 63–73. 10.1016/j.jclinepi.2019.02.016 30878639 10.1016/j.jclinepi.2019.02.016 PMC6547017

[CR36] Nugter, M. A., Hermens, M. L. M., Robbers, S., Van Son, G., Theunissen, J., & Engelsbel, F. (2017). Use of outcome measurements in clinical practice: How specific should one be? *Psychotherapy Research,**29*(4), 432–444. 10.1080/10503307.2017.1408975 29199522 10.1080/10503307.2017.1408975

[CR37] Rameckers, S. A., Verhoef, R. E. J., Grasman, R. P. P. P., Cox, W. R., van Emmerik, A. A. P., Engelmoer, I. M., & Arntz, A. (2021). Effectiveness of psychological treatments for borderline personality disorder and predictors of treatment outcomes: A multivariate multilevel meta-analysis of data from all design types. *Journal of Clinical Medicine,**10*(23), 5622. 10.3390/jcm10235622 34884324 10.3390/jcm10235622 PMC8658126

[CR38] Rognstad, K., Wentzel-Larsen, T., Neumer, S. P., & Kjøbli, J. (2023). A systematic review and meta-analysis of measurement feedback systems in treatment for common mental health disorders. *Administration and Policy in Mental Health,**50*(2), 269–282. 10.1007/s10488-022-01236-9 36434313 10.1007/s10488-022-01236-9 PMC9931854

[CR39] Rossi, G., Debast, I., & Van Alphen, S. P. J. (2017). Measuring personality functioning in older adults: Construct validity of the Severity Indices of Personality Functioning-Short Form (SIPP-SF). *Aging & Mental Health,**21*(7), 703–711. 26923265 10.1080/13607863.2016.1154012

[CR40] Schibbye, P., Ghaderi, A., Ljótsson, B., Hedman, E., Lindefors, N., Rück, C., & Kaldo, V. (2014). Using early change to predict outcome in cognitive behaviour therapy: Exploring timeframe, calculation method, and differences of disorder-specific versus general measures. *PLoS ONE,**9*(6), e100614. 10.1371/journal.pone.0100614 24959666 10.1371/journal.pone.0100614 PMC4069083

[CR42] Stekhoven, D. J. (2022). Using the missForest Package. Update: Version1.5. https://cran.r-project.org/web/packages/missForest/vignettes/missForest_1.5.pdf

[CR43] Stekhoven, D. J., & Bühlmann, P. (2012). MissForest—non-parametric missing value imputation for mixed-type data. *Bioinformatics,**28*(1), 112–118. 10.1093/bioinformatics/btr597 22039212 10.1093/bioinformatics/btr597

[CR44] Storebø, O. J., Stoffers-Winterling, J. M., Völlm, B. A., Kongerslev, M. T., Mattivi, J. T., Jørgensen, M. S., & Simonsen, E. (2020). Psychological therapies for people with borderline personality disorder. *Cochrane Database of Systematic Reviews*. 10.1002/14651858.CD012955.pub2 32368793 10.1002/14651858.CD012955.pub2 PMC7199382

[CR45] Sweeney, L. (2002). k-anonymity: A model for protecting privacy. *International Journal of Uncertainty, Fuzziness and Knowledge-Based Systems,**10*(05), 557–570. 10.1142/S0218488502001648 10.1142/S0218488502001648

[CR46] Swift, J. K., & Greenberg, R. P. (2014). A treatment by disorder meta-analysis of dropout from psychotherapy. *Journal of Psychotherapy Integration,**24*(3), 193–207. 10.1037/a0037512 10.1037/a0037512

[CR47] Tiemens, B., Bocker, K., & Kloos, M. (2016). Prediction of treatment outcome in daily generalized mental healthcare practice: First steps towards personalized treatment by clinical decision support. *European Journal for Person Centered Healthcare.,**4*(1), 24–32. 10.5750/ejpch.v4i1.1044

[CR48] Tiemens, B. G., Kramer, M. I., Kloos, M. W., & Spijker, J. (2020). ROM vroeg in de behandeling en specifiek; observationeel onderzoek naar generieke en specifieke vragenlijsten bij meten van vroege verandering bij depressiebehandeling. [ROM early treatment and specific; observational research of generic and specific questionnaires in measuring early change in depression treatment]. *Tijdschrift voor Psychiatrie*, 121–130. 32141519

[CR49] Timman, R., de Jong, K., & de Neve-Enthoven, N. (2017). Cut-off scores and clinical change indices for the Dutch Outcome Questionnaire (OQ-45) in a large sample of normal and several psychotherapeutic populations. *Clinical Psychology & Psychotherapy,**24*(1), 72–81. 10.1002/cpp.1979 26497324 10.1002/cpp.1979

[CR53] van der Mheen, M., ter Mors, L. M., van den Hout, M. A., & Cath, D. C. (2018). Routine outcome monitoring bij de behandeling van angststoornissen: Diagnosespecifieke versus generieke meetinstrumenten. [Routine outcome monitoring in the treatment of anxiety disorders: Diagnosis-specific versus generic measurement tools]. *Tijdschrift voor Psychiatrie,**60*(1), 11–19. 29341052

[CR50] Verheul, R., Andrea, H., Berghout, C. C., Dolan, C., Busschbach, J. J. V., van der Kroft, P. J. A., Bateman, A. W., & Fonagy, P. (2008). Severity Indices of Personality Problems (SIPP-118): Development, factor structure, reliability, and validity. *Psychological Assessment,**20*(1), 23–34. 10.1037/1040-3590.20.1.23 18315396 10.1037/1040-3590.20.1.23

[CR51] Walfish, S., McAlister, B., O’Donnell, P., & Lambert, M. J. (2012). An investigation of self-assessment bias in mental health providers. *Psychological Reports,**110*(2), 639–644. 10.2466/02.07.17.PR0.110.2.639-644 22662416 10.2466/02.07.17.PR0.110.2.639-644

