# Supplementary material for: Early Change as a Predictor of Treatment Outcome in Patients with a Personality Disorder
Source: Adm Policy Ment Health. 2024 Aug 7;51(5):780–91. doi: 10.1007/s10488-024-01401-2 (PMC11379748; doi:10.1007/s10488-024-01401-2)
Supplement: Supplementary file 1 — Supplementary file1 (DOCX 34 KB) [file 10488_2024_1401_MOESM1_ESM.docx]

**Supplementary material**

**Missing data analysis**

Table 1. shows the percentage of missing data per measure at each measurement point. The missing data was analyzed using the VIM R package (Kowarik & Templ, 2016) by creating a dummy variable (0 = missing, 1 = not missing) and performing chi-square tests to see whether scores on variables were related to missingness.

Age categories significantly differed on prevalence of missingness in OQ-45 scores (χ^2^(4, N = 294) = 10.136, *p* = .038), where patients in the age category of 56 years and older were more likely to miss OQ-45 post-treatment scores. Also, treatment frequency seemed to be related to missingness on the measures. The 2-day treatment group had more missing data on SIPP (χ^2^ (1, N = 602) = 80.814, *p* < .001), GAPD pre-treatment scores (χ^2^ (1, N = 570) = 63.168, *p* < .001) and SIPP intermediate scores (χ^2^ (1, N = 614) = 4.452, *p* = .035), whereas the 4-day treatment group had more missing data on the OQ-45 pre-treatment scores (χ^2^ (1, N = 647) = 75.631, *p* < .001) and GAPD post-treatment scores (χ^2^ (1, N = 263) = 3.942, *p* = .047). No significant differences were found regarding treatment type. The missing data did not seem to be related to the scores on the measures and thus related to the observed but not the unobserved data, which indicates that the data were Missing at random (MAR; Mack et al., 2018).

Table 1. Percentage missing data per measure

| Questionnaire | Measurement | Missing data *N* (%) |
| --- | --- | --- |
| GAPD | Pre-treatment | 271 (32.2 %) |
|  | Intermediate 1 | 566 (67.3 %) |
|  | Intermediate 2 | 548 (65.2 %) |
|  | Post-treatment | 578 (68.7%) |
| OQ-45 SD | Pre-treatment | 194 (23.1%) |
|  | Intermediate 1 | 519 (61.7%) |
|  | Intermediate 2 | 524 (62.3 %) |
|  | Post-treatment | 547 (65.0 %) |
| SIPP | Pre-treatment | 239 (28.4 %) |
|  | Intermediate 1 | 534 (63.5 %) |
|  | Intermediate 2 | 534 (63.5 %) |
|  | Post-treatment | 563 (66.9 %) |

*Note.* Data of 162 (19.3%) participants were complete. GAPD: General Assessment of Personality Disorder. OQ-45.2, SD: Outcome Questionniare-45.2, Symptomatic Distress scale. SIPP: Severity Indices of Personality Problems.

Tabel 2. Descriptives of the scores on the measures in the original and imputed data

| Original data Imputed data | | | | |
| --- | --- | --- | --- | --- |
|  | *M* | *SD* | *M* | *SD* |
| GAPD pre-treatment | 2.8 | .5 | 2.8 | .5 |
| GAPD intermediate measurement 1 | 2.9 | .5 | 2.9 | .5 |
| GAPD intermediate measurement 2 | 2.9 | .6 | 2.9 | .5 |
| GAPD post-treatment | 2.5 | .7 | 2.6 | .6 |
| OQ-45 SD pre-treatment | 59.9 | 12.4 | 59.7 | 11.5 |
| OQ-45 SD intermediate measurement 1 | 57.0 | 13.3 | 57.2 | 13.1 |
| OQ-45 SD intermediate measurement 2 | 56.1 | 13.3 | 57.5 | 12.0 |
| OQ-45 SD post-treatment | 46.0 | 19.1 | 48.6 | 15.9 |
| SIPP SC pre-treatment | 37.9 | 10.2 | 37.9 | 9.4 |
| SIPP SA pre-treatment | 43.9 | 11.3 | 43.9 | 10.5 |
| SIPP R pre-treatment | 40.7 | 12.9 | 40.8 | 12.0 |
| SIPP RF pre-treatment | 33.4 | 10.0 | 33.6 | 9.1 |
| SIPP II pre-treatment | 28.0 | 8.3 | 27.9 | 7.6 |
| SIPP SC intermediate measurement 1 | 41.0 | 10.9 | 42.0 | 10.4 |
| SIPP SC intermediate measurement 2 | 42.2 | 10.9 | 42.0 | 10.2 |
| SIPP SA intermediate measurement 1 | 44.6 | 11.6 | 44.8 | 11.2 |
| SIPP SA intermediate measurement 2 | 45.4 | 11.1 | 45.6 | 10.3 |
| SIPP R intermediate measurement 1 | 40.7 | 13.3 | 41.4 | 12.1 |
| SIPP R intermediate measurement 2 | 40.2 | 12.2 | 39.7 | 11.7 |
| SIPP RF intermediate measurement 1 | 34.9 | 10.4 | 34.7 | 9.5 |
| SIPP RF intermediate measurement 2 | 34.4 | 9.9 | 34.6 | 8.8 |
| SIPP II intermediate measurement 1 | 28.8 | 9.6 | 28.5 | 9.3 |
| SIPP II intermediate measurement 2 | 28.8 | 10.2 | 28.2 | 8.8 |
| SIPP SC post-treatment | 46.3 | 10.8 | 45.5 | 8.5 |
| SIPP SA post-treatment | 48.8 | 11.5 | 48.0 | 9.4 |
| SIPP R post-treatment | 45.4 | 12.7 | 44.0 | 10.9 |
| SIPP RF post-treatment | 40.8 | 12.3 | 39.0 | 9.8 |
| SIPP II post-treatment | 37.1 | 13.1 | 34.4 | 10.6 |

*Note.* The scores on the SIPP are t-scores. SC = Self-control; SA = Social attunement, R = Responsibility; RF = Relational functioning; II= Identity integration.

|  | 1. | 2. | 3. | 4. | 5. | 6. | 7. | 8. | 9. | 10. | 11. | 12. | 13. | 14. | 15. | 16. | 17. | 18. | 19. | 20. | 21. | 22. | 23. | 24. | 25. | 26. | 27. |
| --- | --- | --- | --- | --- | --- | --- | --- | --- | --- | --- | --- | --- | --- | --- | --- | --- | --- | --- | --- | --- | --- | --- | --- | --- | --- | --- | --- |
| 1. SIPP SC pre |  |  |  |  |  |  |  |  |  |  |  |  |  |  |  |  |  |  |  |  |  |  |  |  |  |  |  |
| 2. SIPP SA pre | .66 |  |  |  |  |  |  |  |  |  |  |  |  |  |  |  |  |  |  |  |  |  |  |  |  |  |  |
| 3. SIPP R pre | .47 | .43 |  |  |  |  |  |  |  |  |  |  |  |  |  |  |  |  |  |  |  |  |  |  |  |  |  |
| 4. SIPP RF pre | .31 | .39 | .24 |  |  |  |  |  |  |  |  |  |  |  |  |  |  |  |  |  |  |  |  |  |  |  |  |
| 5. SIPP II pre | .53 | .32 | .31 | .58 |  |  |  |  |  |  |  |  |  |  |  |  |  |  |  |  |  |  |  |  |  |  |  |
| 6. OQ-45 SD pre | -.39 | -.21 | -.12 | -.40 | -.66 |  |  |  |  |  |  |  |  |  |  |  |  |  |  |  |  |  |  |  |  |  |  |
| 7. GAPD pre | -.56 | -.47 | -.43 | -.63 | -.74 | .51 |  |  |  |  |  |  |  |  |  |  |  |  |  |  |  |  |  |  |  |  |  |
| 8. SIPP SC IM 1 | .81 | .58 | .40 | .25 | .37 | -.26 | -.45 |  |  |  |  |  |  |  |  |  |  |  |  |  |  |  |  |  |  |  |  |
| 9. SIPP SC IM 2 | .81 | .58 | .40 | .25 | .37 | -.26 | -.44 | 1.00 |  |  |  |  |  |  |  |  |  |  |  |  |  |  |  |  |  |  |  |
| 10. SIPP SA IM 1 | .56 | .80 | .35 | .25 | .21 | -.13 | -.36 | .67 | .66 |  |  |  |  |  |  |  |  |  |  |  |  |  |  |  |  |  |  |
| 11. SIPP SA IM 1 | .56 | .79 | .37 | .24 | .20 | -.12 | -.36 | .67 | .67 | .99 |  |  |  |  |  |  |  |  |  |  |  |  |  |  |  |  |  |
| 12. SIPP R IM 1 | .38 | .40 | .87 | .24 | .30 | -.12 | -.42 | .42 | .41 | .40 | .41 |  |  |  |  |  |  |  |  |  |  |  |  |  |  |  |  |
| 13. SIPP R IM 2 | .39 | .41 | .86 | .25 | .30 | -.13 | -.43 | .42 | .42 | .41 | .42 | 1.00 |  |  |  |  |  |  |  |  |  |  |  |  |  |  |  |
| 14. SIPP RF IM 1 | .18 | .24 | .17 | .83 | .50 | -.35 | -.53 | .21 | .21 | .21 | .20 | .25 | .26 |  |  |  |  |  |  |  |  |  |  |  |  |  |  |
| 15. SIPP RF IM 2 | .18 | .24 | .16 | .82 | .50 | -.35 | -.53 | .21 | .21 | .21 | .21 | .25 | .26 | .99 |  |  |  |  |  |  |  |  |  |  |  |  |  |
| 16. SIPP II IM 1 | .31 | .15 | .07* | .50 | .75 | -.61 | -.56 | .30 | .30 | .13 | .12 | .17 | .18 | .59 | .61 |  |  |  |  |  |  |  |  |  |  |  |  |
| 17. SIPP II IM 2 | .31 | .15 | .07* | .50 | .75 | -.61 | -.56 | .29 | .30 | .14 | .13 | .17 | .18 | .60 | .62 | .99 |  |  |  |  |  |  |  |  |  |  |  |
| 18. OQ-45 SD IM 1 | -.27 | -.12 | -.02 | -.37 | -.58 | .73 | .42 | -.27 | -.27 | -.11 | -.11 | -.10 | -.11 | -.45 | -.46 | -.76 | -.76 |  |  |  |  |  |  |  |  |  |  |
| 19. OQ-45 SD IM 2 | -.27 | -.12 | -.02 | -.37 | -.59 | .73 | .42 | -.28 | -.28 | -.13 | -.13 | -.11 | -.12 | -.45 | -.47 | -.78 | -.78 | .98 |  |  |  |  |  |  |  |  |  |
| 20. GAPD IM 1 | -.49 | -.46 | -.35 | -.58 | -.64 | .47 | .74 | -.51 | -.52 | -.46 | -.47 | -.45 | -.46 | -.63 | -.64 | -.68 | -.68 | .57 | .58 |  |  |  |  |  |  |  |  |
| 21. GAPD IM 2 | -.49 | -.45 | -.36 | -.58 | -.64 | .46 | .74 | -.51 | -.51 | -.45 | -.46 | -.45 | -.47 | -.63 | -.64 | -.68 | -.68 | .56 | .58 | .99 |  |  |  |  |  |  |  |
| 22. SIPP SC post | .69 | .59 | .35 | .33 | .40 | -.35 | -.48 | .75 | .75 | .61 | .61 | .39 | .40 | .27 | .27 | .34 | .35 | -.38 | -.38 | -.57 | -.55 |  |  |  |  |  |  |
| 23. SIPP SA post | .57 | .79 | .35 | .34 | .29 | -.26 | -.43 | .61 | .61 | .84 | .84 | .40 | .40 | .27 | .27 | .23 | .24 | -.24 | -.24 | -.53 | -.51 | .78 |  |  |  |  |  |
| 24. SIPP R post | .42 | .46 | .75 | .34 | .37 | -.21 | -.49 | .44 | .44 | .43 | .45 | .85 | .86 | .34 | .34 | .27 | .27 | -.22 | -.23 | -.54 | -.54 | .59 | .56 |  |  |  |  |
| 25. SIPP RF post | .21 | .29 | .21 | .73 | .49 | -.42 | -.54 | .25 | .25 | .26 | .25 | .30 | .31 | .78 | .78 | .55 | .56 | -.51 | -.50 | -.62 | -.61 | .50 | .47 | .51 |  |  |  |
| 26. SIPP II post | .26 | .19 | .11 | .45 | .59 | -.56 | -.50 | .27 | .27 | .17 | .17 | .20 | .21 | .48 | .48 | .67 | .67 | -.67 | -.66 | -.58 | -.56 | .59 | .42 | .46 | .77 |  |  |
| 27. OQ-45 SD post | -.43 | -.43 | -.30 | -.54 | -.57 | .51 | .63 | -.43 | -.44 | -.41 | -.41 | -.39 | -.40 | -.54 | -.54 | -.57 | -.58 | .57 | .56 | .73 | .72 | -.73 | -.64 | -.63 | -.82 | -.87 |  |
| 28. GAPD post | -.29 | -.22 | -.10 | -.38 | -.50 | .62 | .42 | -.29 | -.29 | -.21 | -.21 | -.18 | -.19 | -.40 | -.41 | -.58 | -.58 | .74 | .72 | .53 | .51 | -.61 | -.46 | -.41 | -.69 | -.91 | .83 |

Table 3. Correlations between measures at all measurement points *Note.* All correlations *p* <.01 level, except: * *p* < .05 level. IM = Intermediate measurement. SC = Self-control; SA = Social attunement, R = Responsibility; RF = Relational functioning; II= Identity integration.
